# Entomological consequences and toxicological detection of synthetic cannabinoid receptor agonists (SCRAs) in necrophagous larvae (Diptera: Calliphoridae)

**DOI:** 10.1007/s00414-025-03688-8

**Published:** 2026-01-02

**Authors:** Chloé A. K. Blavier, Martin H. Villet, Annette Zschiesche, Volker Auwärter, Matthias Graw, Christoph Geffert, Olwen C. Groth

**Affiliations:** 1 Institute of Forensic Medicine, Ludwig-Maximilians-Universität in Munich, Nussbaumstrasse 26, 80336 Munich, Germany; 2 Polytech Nice Sophia Engineering School – University Côte d’Azur, 06903 Sophia Antipolis, France; 3 Department of Zoology & Entomology, Rhodes University, Makhanda, 6140 South Africa; 4 Institute of Forensic Medicine, Forensic Toxicology, Medical Center, Faculty of Medicine, University of Freiburg, University of Freiburg, Albertstrasse 9, 79104 Freiburg, Germany; 5 Hermann Staudinger Graduate School, University of Freiburg, Hebelstrasse 27, 79104 Freiburg, Germany; 6 Labor Staber, Bremer Strasse 9, 01665 Klipphausen, Germany

**Keywords:** 5-Fluoro-ADB (5F-ADB, 5F-MDMB-PINACA), ADB-BUTINACA, MDMB-4en-PINACA, Minimum post-mortem interval (PMI_min_) estimation, New psychoactive substances (NPSs), Synthetic cannabinoids

## Abstract

Forensic entomologists estimate minimum post-mortem intervals (PMI_min_) by measuring the development of larvae on decomposing bodies. Entomotoxicology assumes significance when the deceased had ingested drugs, as these may alter insect development and consequently the accuracy of PMI_min_ estimations. 5F-ADB, ADB-BUTINACA, and MDMB-4en-PINACA are potent synthetic cannabinoid receptor agonists and drugs of abuse, linked to human intoxications and deaths. We investigated the impact of 5F-ADB on the development of *Lucilia sericata* (Meigen, 1826) (Diptera: Calliphoridae) larvae and developed a method to detect 5F-ADB, ADB-BUTINACA, MDMB-4en-PINACA, and their metabolites in larvae by liquid chromatography-mass spectrometry. Neonate larvae were exposed to 5F-ADB concentrations that reflect those in human post-mortem specimens. At precise intervals, larvae were killed in near-boiling water, dried, and then their size measured, followed by toxicological analysis. ANOVA demonstrated that only time (= age) significantly affected larval length and mass (through growth). Drug concentrations typically found in post-mortem peripheral blood did not significantly affect larval development, suggesting that 5F-ADB in a corpse would not influence PMI_min_ estimation with *L. sericata*. 5F-ADB was detected in trace amounts in larvae that were exposed to post-mortem peripheral blood concentrations. Most larvae from higher concentration treatments contained 5F-ADB levels above the limit of detection of 0.25 µg/kg, but not all samples tested positive. Post-feeding larvae mostly did not contain detectable levels of 5F-ADB, and metabolites were found in none of the larval samples. Actively feeding *L. sericata* larvae from a corpse may thus be useful to qualitatively identify a prior consumption of 5F-ADB by the deceased.

**Supplementary Information:**

The online version contains supplementary material available at 10.1007/s00414-025-03688-8.

## Introduction

In the field of forensics, estimating the post-mortem interval (PMI) is often essential to death investigations, as it represents the time that has elapsed since a death, which may be crucial evidence. In cases of decomposed bodies, traditional methods may not provide reliable results because body temperature equalises with the surrounding environment (algor mortis) in hours, and rigor and livor mortis are almost as transient and harder to assess [1]. Forensic entomological methods therefore play an important role over longer post-mortem intervals in estimating the minimum PMI (PMI_min_) of decomposed bodies, which refers to the minimum time between death and discovery of the remains [2, 3].

Blowflies (Calliphoridae) are attracted by decomposition odours and promptly lay their eggs on cadavers. First-instar larvae hatch from these eggs, start feeding on the carcass and grow, which allows forensic entomologists to estimate the PMI_min_ from the size (length or mass) of the oldest immature insects on and around the corpse [3, 4]. The life cycles of various necrophagous insect species are well-documented, enabling forensic entomologists to estimate the timing of egg deposition from the developmental stage of the insects at their discovery [5]. Therefore, entomology is considered one of the most accurate methods to determine the post-mortem interval [6], especially when death occurred more than 72 h ago [3, 7].

The present study is focused on the field of forensic entomotoxicology, a part of which builds on the principles of entomology to examine the effects of toxicants on the development of necrophagous insects [8, 9]. A potential limitation in the entomological estimation of PMIs is that drugs ingested by the deceased before death could alter insect development and lead to inaccurate age estimates [3, 10]. This is not always considered in real casework, partially due to the complex nature of drug-dependent influences on insect growth in true forensic scenarios. Depending on the specific drug in question, larval development can either be accelerated [11], slowed down [12, 13], or remain unaltered [14]. It is imperative to take the first two effects into account to avoid under- or overestimating the time since death.

**Fig. 1:**
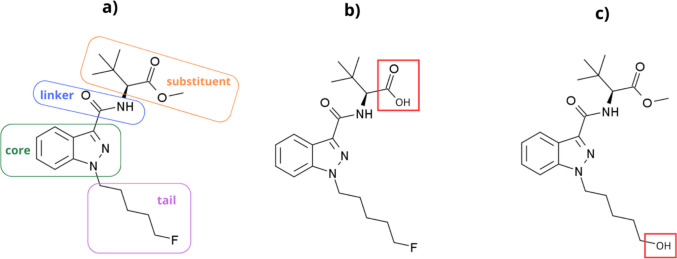
**(a)** Chemical structure of 5F-ADB, consisting of an indazole core, a carboxamide linker, a *tert*-leucinate methyl substituent, and a 5-fluoropentyl tail [16], **(b)** its methyl ester hydrolysis product, 5F-ADB carboxylic acid, and **(c)** its hydrolytic/oxidative defluorination metabolite [19, 22, 23]

Although several drugs from diverse drug classes have been investigated for their effect on necrophagous insect development [8–10], the synthetic cannabinoid receptor agonists (SCRAs) remain unexplored in this regard. SCRAs represent the largest group of new psychoactive substances (NPSs) currently monitored by the European Union Drugs Agency (EUDA) through the EU Early Warning System. To date, the European drug market has witnessed nearly 250 different SCRAs that vary in chemical composition [15], most of which share a common chemical skeleton consisting of a core structure to which a linker with substituent and tail derivative are attached [16] (Fig. 1a). These man-made chemicals are mostly characterised by their high affinity as agonists for the CB_1_ and CB_2_ cannabinoid receptors. They are often abused for their psychotropic effects as a substitute for the natural phytocannabinoid Δ^9^-tetrahydrocannabinol (THC), typically exhibiting greater potency than THC [17–19]. The relative potencies among these substances themselves also vary significantly, which is attributable to their high structural diversity [16, 20, 21].

Although the prevalence of SCRA-related intoxications and deaths have significantly decreased since the introduction of generic laws [24–26], some of these drugs still pose a relevant health risk [27]. Among the various chemical structures available, 5F-ADB (5F-MDMB-PINACA, Fig. 1a) is not only one of the most prevalent synthetic cannabinoids on the European, US, and Brazilian drug markets at present [15, 28, 29], but also one of the most potent in its class [22]. Case studies have reported acute intoxications with 5F-ADB, several of which resulted in death [24, 30–34]. Recently, 5F-ADB has even experienced a resurgence on the illicit drug market [35] following a decline in intoxication cases [24].

5F-ADB undergoes extensive ester hydrolysis in humans to form the carboxylic acid derivative (methyl 2-[1-(5-fluoropentyl)−1*H*-indazole-3-carboxamido]−3,3-dimethyl-butanoic acid, Fig. 1b) as a major metabolite. Other biotransformation pathways in humans include hydrolytic/oxidative defluorination of the parent compound, creating 2-[[1-(5-hydroxypentyl)−1*H*-indazole-3-carbonyl]amino]−3,3-dimethyl butanoate (Fig. 1c) [23, 36].

**Fig. 2:**
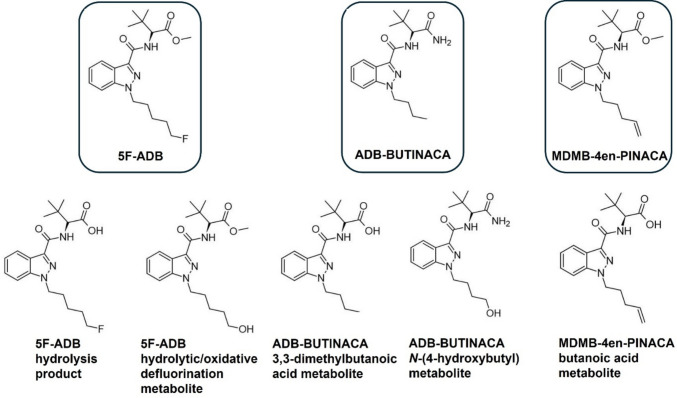
Analytes included in the LC–MS/MS method for their detection in larvae

Additional to the effect of drugs on necrophagous insect development, another focal point of forensic entomotoxicology is their use as proxy samples for toxicological analysis [8, 37]. Insects associated with corpses in advanced phases of decomposition may be the sole source available to identify prior drug consumption by the deceased [38–40]. The toxicological analysis of insects could even provide additional information to that obtained from traditional methods [41, 42]. The presence of some SCRAs has been identified in larvae from post-mortem cases [41], indicating the potential for necrophagous insects to accumulate these substances. To the best of the authors’ knowledge, pre-analytical methods for the successful detection of SCRAs in larvae have not yet been published. Considering their role in intoxication cases [27], the development of a method for such an analysis was a primary aim of this work. For this purpose, 5F-ADB, two further potent and prevalent SCRAs, ADB-BUTINACA (ADB-BINACA, ADMB-BINACA, *N*-[1-amino-3,3-dimethyl-1-oxobutan-2-yl]−1-butyl-1*H*-indazole-3-carboxamide), and MDMB-4en-PINACA (methyl 3,3-dimethyl-2-[1-(pent-4-en-1-yl)−1*H*-indozole-3-carboxamido] butanoate) [29, 43–46], as well as their metabolites were included as target analytes (Fig. 2).

The blowfly species *Lucilia sericata* (Meigen, 1826) (Diptera: Calliphoridae) was selected as model insect because it is one of the most globally distributed necrophagous species [10, 47, 48]. Furthermore, the life cycle, genome, and transcriptome of *L. sericata* are well-documented [49–52], and numerous case reports have demonstrated its efficacy in inferring the PMI [53–55], thus establishing its forensic value.

In the present study, *Lucilia sericata* was used to evaluate the effect of 5F-ADB on necrophagous larval development and PMI_min_ estimation. Furthermore, the potential of entomotoxicological methods to identify prior consumption of this substance by a deceased person was evaluated through the toxicological analysis of *L. sericata* larvae that had been exposed to different concentrations of the drug for varying periods of time. For this purpose, an analytical method was developed and optimised to effectively extract and detect SCRAs and their metabolites from larvae that had been exposed to them.

## Materials and methods

Two experiments were performed, during which larvae were exposed to lower and higher concentrations of 5F-ADB.

To establish a colony of *L. sericata* for the first in vitro experiment, which also served to investigate drug influence on insect development, pupae of *L. sericata* were purchased from a commercial supplier (TerraristikShop.net, Herzogenaurach, Germany). Seventy pupae were placed in each of five rearing cages, each measuring 35 × 21 × 21 cm, and maintained at room temperatures. The species of the first emerging flies was confirmed morphologically using a taxonomic key for European blowflies [56]. Moistened sugar cubes and water in the form of soaked paper towels were provided ad libitum. Two large cups of raw, minced pork meat were introduced into each cage every evening to facilitate egg maturation and oviposition. Hatching larvae were either bred to the third instar to provide larval homogenate for toxicological method development (see Sect. 2.2) or bred to the end of the third generation for the first in vitro growth experiment.

For the second set of in vitro experiments, wild adults of *L. sericata* were collected outside the Institute of Forensic Medicine, Munich, using traps baited with raw sheep liver. Adult flies were maintained at ambient temperatures in rearing cages, with a maximum of two to three adults per cage. Raw, minced pork meat was introduced into each cage to encourage oviposition. After oviposition, adult flies were killed for morphological confirmation of the species [56], and only larvae hatching from egg clusters from *L. sericata* adults were used for further experiments.

### In vitro* experiments*

#### *Meta-analysis of 5F-ADB concentrations in post-mortem peripheral blood to serve as a guideline to determine concentrations for *In vitro* experiment I*

**Fig. 3:**
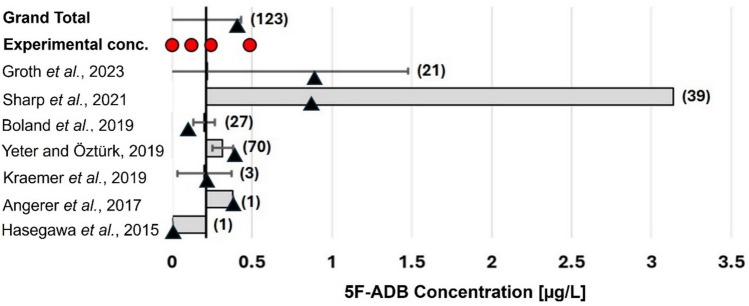
Meta-analysis of seven published studies [24, 32, 33, 36, 57–59] of 5F-ADB concentrations in femoral blood of deceased persons, sampled at autopsy, indicating the median study effect (box)^a^, 95% confidence interval (CI_95_: error bars), mean (▲, triangles), and sample size (in brackets to right). 5F-ADB concentrations used for In vitro experiment I are indicated by red circles. (The raw data from Sharp et al*.* [58] were not available to be incorporated into the calculations)

^a^Median of the individual study relative to that of the overall median.

Meta-analysis of post-mortem concentrations of 5F-ADB in peripheral blood was performed on an extensive literature review of intoxication cases with synthetic cannabinoids. Cases from seven published studies [24, 32, 33, 36, 57–59] were included, irrespective of post-mortem interval. Publications with available raw data (123 relevant cases) were used to calculate an overall median (0.21 µg/L) and mean concentration (0.41 µg/L) for 5F-ADB in post-mortem peripheral blood, which were used to define 5F-ADB concentrations for the first in vitro experiment using spiked meat (Fig. 3).

Based on the results obtained from the meta-analysis, 0.12 µg/kg, 0.24 µg/kg, and 0.48 µg/kg were chosen as lower, intermediate, and upper concentrations, respectively, for the treatments of In vitro experiment I (Fig. 3). Food medium without the drug (0 µg/kg) served as control.

#### *Literature review of 5F-ADB concentrations in post-mortem solid tissues to serve as a guideline to determine concentrations for *In vitro* experiment II*

A second set of in vitro experiments were conducted to reflect 5F-ADB concentrations in solid human post-mortem tissues. For this, concentration data from three publications [24, 32, 60] could be employed as a guideline to determine 5F-ADB concentrations in the food medium (Table 1). Based on these data, 0 µg/kg, 0.48 µg/kg, 1.8 µg/kg, and 7.7 µg/kg were chosen as control, lower, intermediate, and upper concentration treatments for In vitro experiment II.

**Table 1:** Post-mortem concentrations of 5F-ADB in solid human tissues, grouped according to the lower, intermediate, and higher concentration ranges

Concentration reported [µg/kg]	Calculated mean concentration	Tissue type	Reference	
< 0.1^a^	Low *Range:* *0.1–0.5 µg/kg*	Liver	Neukamm et al., 2024	[60]
0.1^a^–0.5^b^		Liver	Hasegawa et al*.*, 2015	[32]
0.1^a^–0.5^b^		Lung	Hasegawa et al*.*, 2015	[32]
0.1^a^–0.5^b^		Skeletal muscle	Hasegawa et al*.*, 2015	[32]
0.1^a^–0.5^b^		Kidney	Hasegawa et al*.*, 2015	[32]
0.98	Intermediate *1.8 µg/kg*	Kidney	Neukamm et al., 2024	[60]
1.17 ± 0.016		Spleen	Hasegawa et al*.*, 2015	[32]
1.61 ± 0.042		Pancreas	Hasegawa et al*.*, 2015	[32]
1.82 ± 0.041		Brain tissue	Hasegawa et al*.*, 2015	[32]
1.90 ± 0.078		Heart tissue	Hasegawa et al*.*, 2015	[32]
3.18 ± 0.084		Stomach contents	Hasegawa et al*.*, 2015	[32]
7.2	High *7.7 µg/kg*	Liver	Groth et al*.*, 2023	[24]
7.95 ± 0.026		Adipose tissue	Hasegawa et al*.*, 2015	[32]

^*a*^*Limit of detection, *^*b*^*Limit of quantification*

#### Preparation of fortified food medium

During growth experiments, limiting the amount of organic solvent in the food matrix is crucial to minimise any additional effect (*i.e.* other than the drug itself) on insect development. At the same time, drug solubility should be maintained for optimal homogeneity throughout the food source. To achieve this, a 1:4 mixture of ethanol (96%, Roti®Cell, Roth, Karlsruhe, Germany) and Dulbecco’s phosphate-buffered saline (DPBS, pH 7.0 ± 0.2, Roti®Cell, Roth, Karlsruhe, Germany) was prepared, based on the 5F-ADB-manufacturer’s specifications of its solubility. The 1:4 mixture of ethanol:DPBS served as solvent for a 120 µg/mL stock solution of (R)−5-fluoro-ADB (5F-ADB) (≥ 98%, Cayman Chemical, Ann Arbor, MI, USA), which was diluted in the same 1:4 mixture to afford 1.2 µg/mL and 0.12 µg/mL working solutions for fortification.

Raw, lean, minced pork meat, obtained from a commercial butcher, was used as food substrate for both in vitro experiments. Minced meat was divided into four batches of 240 g each for In vitro experiment I, and four batches of 120 g each for In vitro experiment II. Each batch was fortified with a total volume of 2 mL (experiment I) or 1 mL (experiment II), comprising the required volume of 5F-ADB solution and a drug-free 1:4 mixture of ethanol:DPBS to obtain the chosen experimental concentrations in meat. The control treatments (0 µg/kg) were spiked with 2 mL and 1 mL, respectively, of the drug-free 1:4 ethanol:DPBS mixture. The concentration treatments for In vitro experiment I, *i.e.* 0.12 µg/kg, 0.24 µg/kg, and 0.48 µg/kg were fortified with 240 µL, 480 µL, and 960 µL of the 0.12 µg/mL 5F-ADB working solution and 1760 µL, 1520 µL, and 1040 µL of the drug-free mixture. Similarly, the following volumes were each diluted to 1 mL with drug-free mixture and spiked to 120 g each of minced pork meat to obtain the concentration treatments 0.48 µg/kg, 1.8 µg/kg, and 7.7 µg/kg for In vitro experiment II: 480 µL of the 0.12 µg/mL working solution, and 180 µL and 865 µL of the 1.2 µg/mL working solutions.

Meat samples were homogenised in a standard kitchen food processor (SilverCrest Nutrition Mixture, Krefeld, Germany) for at least one minute to ensure thorough distribution of the substance within the matrix, taking care to prevent cross-contamination between batches. Each batch of meat was divided into three portions of 80 g each for In vitro experiment I and 40 g each for In vitro experiment II. Each portion was placed into a 100 mL plastic cup (replicates i, ii, and iii for each concentration treatment).

#### Exposing L. sericata larvae to different concentrations of 5F-ADB

For each of the two in vitro experiments, four plastic containers (18 × 11 × 12.5 cm) were prepared for each of the four different treatment concentrations, each container holding some straw for pupariation after the post-feeding stage and a datalogger (FreeTec V2, Munich, Germany) to record temperature and humidity.

- (i) In vitro experiment I

**Fig. 4:**
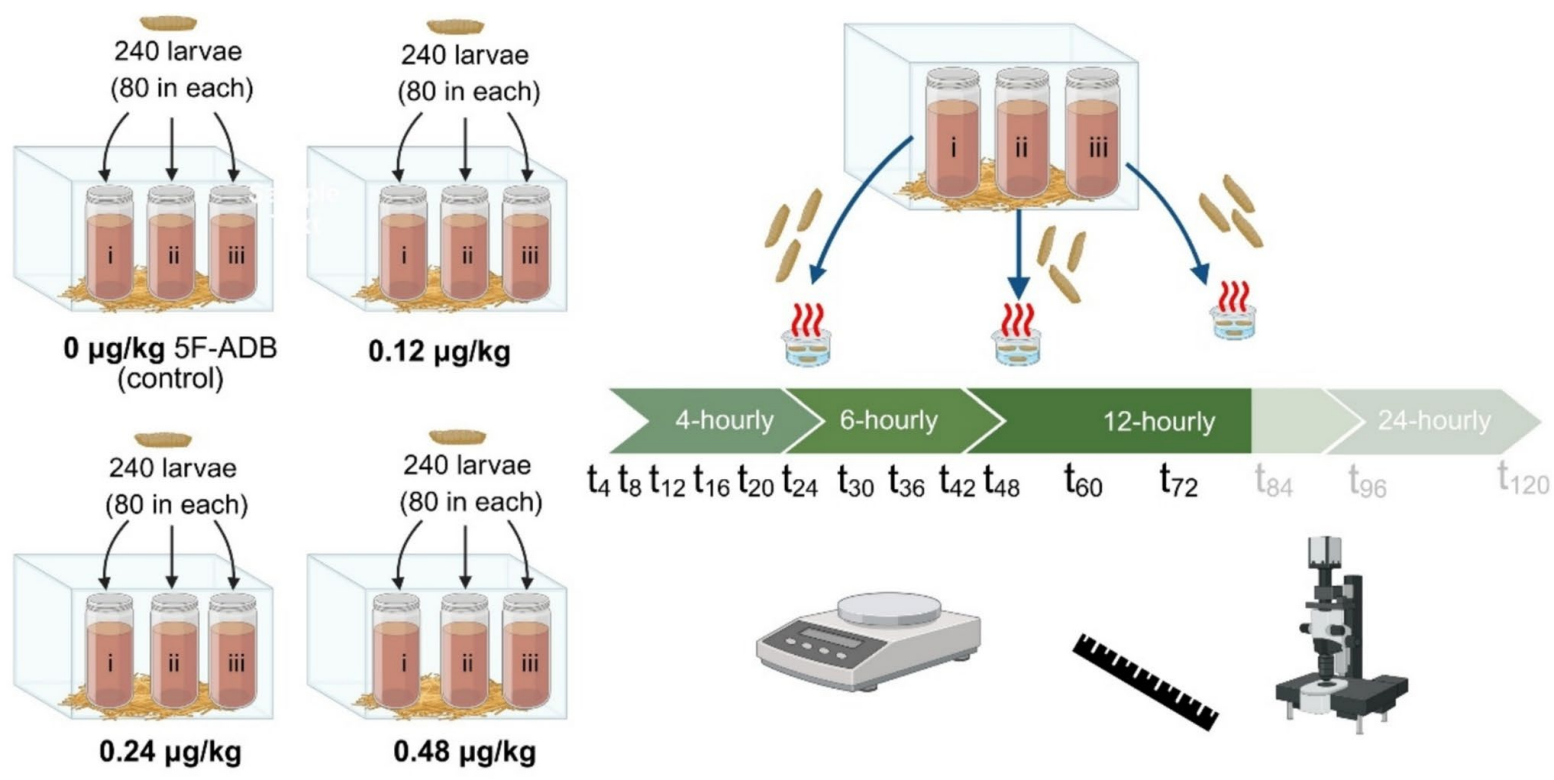
Study design for the treatment and sampling of larvae from the different 5F-ADB treatment regimens from In vitro experiment I (Created with BioRender.com [61]). Only larvae from the 0.24 µg/kg treatment survived after 72 h of exposure

Of third-generation insects from the established colony, 80 neonate larvae were carefully moved into each cup of meat with a moist brush. The three cups with the same drug concentration and larvae (replicates i, ii, and iii in Fig. 4) were placed into the bigger, plastic containers. Plastic containers were covered with nylon nets (Fig. 4) before simultaneous incubation of all four containers in a Memmert IPP 200 incubator (Schwabach, Germany) at 25(± 0.5)℃ and relative humidity (RH) of 70(± 10)%.

Three larvae per cup (i, ii, and iii) and concentration treatment were randomly sampled at specific intervals. Sampling was conducted at 4-hourly intervals for the first 24 h (t_4_, t_8_, t_12_, t_16_, t_20_, and t_24_), then at 6-hourly intervals for the subsequent 24 h (t_30_, t_36_, t_42_, and t_48_), and, where available, at 12-hourly intervals for the subsequent 48 h (t_60_, t_72_, t_84_, and t_96_) (Fig. 4).

- (ii) In vitro experiment II

By applying a moist brush, thirty neonate larvae were carefully moved into each cup, each containing 40 g of minced meat. Analogous to In vitro experiment I (Fig. 4), the three cups with the same drug concentration and larvae were placed into the bigger, plastic containers (*e.g.* replicates i, ii, and iii of the 0.48 µg/kg treatment were placed in the same, bigger container). The containers were each covered with a nylon net, and all treatment concentrations were incubated simultaneous in a Memmert IPP 200 incubator (Schwabach, Germany) at 25(± 0.5)℃ and RH of 70(± 10)%.

Starting at 24 h after incubation, three larvae per replicate cup (i, ii, and iii) and concentration treatment were randomly sampled in 12-hourly intervals until the end of the active feeding stage, followed by two sampling events during the post-feeding phase. Nine larvae from each concentration treatment were thus sampled at each of the following intervals after incubation at t_0_: t_24_, t_36_, t_48_, t_60_, t_72_, t_93_, and t_105_.

- (iii) Killing and storage of larvae

After selection, larvae were immediately killed by immersion in water at approximately 90 °C for one minute. This blanching method is the recommended standard for preserving fly larvae in an extended position, thereby facilitating subsequent length measurement [10, 14, 62] and toxicological analysis [14]. Larvae were washed and carefully dried on paper towels and the mass and length of larvae from In vitro experiment I measured directly after that. The mass of individual larvae was determined using a Mettler Toledo MX-5 microbalance (Greifensee, Switzerland) with a resolution of 1 µg. The body length of each larva was recorded to the nearest half millimetre (0.5 mm) under a Zeiss Stemi DV4 stereomicroscope (Carl Zeiss, Oberkochen, Germany) with a standard ruler [8, 62, 63].

All larvae were placed separately according to sampling time and treatment concentration and stored at −20 °C until toxicological analysis.

#### Statistical analysis

The effects of time (age) and dose on larval length and mass were tested with Analysis of Variance (ANOVA), using Statistica version 14.0.0.15 (2020, TIBICO Software Inc., Palo Alto, CA, USA). Initially, a three-way ANOVA was conducted, with time and dose as fixed effects, and beaker as a random effect. The beaker factor and its interactions were not significant (Supplementary Tables S4 and S5) and were thus not considered in subsequent analyses. A two-way crossed ANOVA was then used, with time and dose as fixed effects. Interaction plots for mass and length were generated to assess the effects of different drug concentrations on larval development over time.

### Toxicological analysis

#### Larval sample preparation

Several extraction procedures (including liquid–liquid extraction, protein precipitation, and solid-phase extraction) with varying parameters (*e.g.* pH and solvent composition) were evaluated for their effect on the recovery of SCRAs and their metabolites from larvae, as well as matrix interferences during liquid chromatography-mass spectrometric analysis. The following analytes with purities ≥ 98%, purchased from Cayman Chemical (Ann Arbor, MI, USA), were included in the analytical method: (R)−5F-ADB (*N*-[[1-(5-fluoropentyl)−1*H*-indazol-3-yl]carbonyl]−3-methyl-D-valine methyl ester), the 5F-ADB hydrolysis product (5F-ADB metabolite 7), the 5F-ADB hydrolytic/oxidative defluorination metabolite (5F-ADB metabolite 2), ADB-BUTINACA (*N*-[(1S)−1-(aminocarbonyl)−2,2-dimethylpropyl]−1-butyl-1*H*-indazole-3-carboxamide), the ADB-BUTINACA *N*-(4-hydroxybutyl) metabolite ((S)-*N*-(1-amino-3,3-dimethyl-1-oxobutan-2-yl)−1-(4-hydroxybutyl)−1*H*-indazole-3-carboxamide), the ADB-BUTINACA 3,3-dimethylbutanoic acid metabolite (MDMB-BUTINACA butanoic acid metabolite, *N*-[(1-butyl-1*H*-indazol-3-yl)carbonyl]−3-methyl-L-valine), MDMB-4en-PINACA (3-methyl-*N*-[[1-(4-penten-1-yl)−1*H*-indazol-3-yl]carbonyl]-L-valine methyl ester), and the MDMB-4en-PINACA butanoic acid metabolite ((S)−3,3-dimethyl-2-(1-(pent-4-en-1-yl)−1*H*-indazole-3-carboxamido) butanoic acid) (Fig. 1).

Considering extraction efficiencies and matrix effects, the most promising method was then applied to larvae that were exposed to 5F-ADB during both in vitro experiments to extract 5F-ADB, its ester hydrolysis product, hydrolytic/oxidative defluorination metabolite, and other human metabolites, followed by detection and quantification by LC–MS/MS. Sample extraction was performed as follows: Larvae exposed to different concentrations of 5F-ADB for varying times were pooled to obtain the required mass of approximately 200 mg per sample for extraction. Larvae from each of the concentration treatments 0.12 µg/kg, 0.24 µg/kg, and 0.48 µg/kg from In vitro experiment I were pooled as follows: 4–42 h, 48–60 h, and 72 h. Only larvae from the 0.24 µg/kg treatment survived after 72 h of exposure, which were also subjected to toxicological analysis. For In vitro experiment II, larvae from each of the concentration treatments 0.48 µg/kg, 1.8 µg/kg, and 7.7 µg/kg were pooled as follows: 24–36 h, 48 h, 60 h, 72 h, 93 h (post-feeding), and 105 h (post-feeding). All larvae from the 0 µg/kg treatments served as negative controls during toxicological analysis.

Each pooled sample of approximately 200 mg was weighed in a 2 mL disposable, reinforced Precellys® vial (Bertin Technologies, Montigny-le-Brettonneux, France), to which 200 µL isotonic sodium chloride (NaCl, ≥ 99%, Roth, Karlsruhe, Germany) solution (0.9% *m/v*) in purified water (Milli-Q Millipore filter system, Bedford, MA, USA) and five stainless steel beads (diameter: 2.8 mm) were added. Larvae were subsequently homogenised in a Precellys® 24 tissue homogeniser at 4000 RPM for 90 s.

For extraction, an internal standard (IS) mix of deuterated SCRAs, containing 5F-MDMB-PICA-*d*_*5*_, AB-FUBINACA-*d*_*4*_, AB-PINACA-*d*_*9*_, the AB-PINACA *N*-pentanoic acid metabolite-*d*_*4*_, ADBICA-*d*_*9*_, ADB-PINACA-*d*_*9*_, the JWH-073 *N*-(4-hydroxybutyl) metabolite-*d*_*5*_, JWH-015-*d*_*7*_, the JWH-018 *N*-(5-hydroxypentyl) metabolite-*d*_*5*_, the JWH-073 *N*-butanoic acid metabolite-*d*_*5*_, the JWH-122 *N*-(5-hydroxypentyl) metabolite-*d*_*5*_, JWH-200-*d*_*5*_, the JWH-250 *N*-(4-hydroxypentyl) metabolite-*d*_*5*_, MAM-2201-*d*_*5*_, and RCS-4-*d*_*9*_ was prepared using 10 µg/mL stock solutions of each analogue in acetonitrile (≥ 99.9%, HPLC Plus, Sigma-Aldrich, Steinheim, Germany), resulting in a final concentration of 250 µg/L each. All deuterated analytes were purchased from Cayman Chemical (Ann Arbor, MI, USA) with purities ≥ 98%. The most suitable deuterated internal standard substance was chosen for each of the analytes to compensate for matrix effects, as summarised in Table S1 of the Supporting information.

After the addition of 2 μL deuterated IS mix to the larval homogenate, 500 μL of acetonitrile with 2% ammonium hydroxide (NH_4_OH, ACS reagent, Sigma Aldrich, Steinheim, Germany) was added and the pH adjusted to 12 with 40 µL of a 10 N aqueous solution of sodium hydroxide (NaOH, ≥ 98%, Sigma Aldrich, Steinheim, Germany). Samples were thoroughly mixed for one minute and centrifuged at 6000 RPM for five minutes, followed by a pass-through SPE-protocol for purification. For this, the supernatant was transferred directly onto Oasis® PRiME HLB 3 cc cartridges (Waters GmbH, Eschborn, Germany), which do not require prior conditioning. The sample was slowly eluted under positive pressure, using the Waters Positive Pressure-96 Processor (Waters GmbH, Eschborn, Germany). Following an additional elution step with 250 µL of 2% NH_4_OH in acetonitrile to improve extraction efficiency, the eluate was evaporated to dryness at 37 °C under a stream of nitrogen.

Extracts were reconstituted shortly before analysis. For reconstitution, 100 µL of a 4:1 mixture of Mobile phase A (1% acetonitrile, 0.1% formic acid, and 2 mM ammonium formate in purified water) and Mobile phase B (1% formic acid and 2 mM ammonium formate in acetonitrile) for HPLC analysis was used. All extracts were passed through 0.45 µm VEREX_TM_ regenerated cellulose (RC) filters (Phenomenex®, Aschaffenburg, Germany) to remove undissolved particles before liquid chromatography-mass spectrometric analysis.

#### Liquid chromatography tandem mass spectrometry (LC–MS/MS)

LC–MS/MS analysis was performed on a Dionex Ultimate 3000 UHPLC system (Thermo Fisher, Dreieich, Germany), coupled to a QTrap 6500 (Sciex, Darmstadt, Germany) and equipped with an electrospray ionisation (ESI) source, operated in positive ionisation mode. Details of the MRM method are summarized in Table S1 of the Supporting information. All data acquisition and processing were performed using Analyst (ver. 1.6, Sciex, Darmstadt, Germany).

Details of the instrumental conditions are published elsewhere [46]. In brief, the oven and autosampler were set to 40 °C and 10 °C, respectively. Chromatographic separation was achieved on a Kinetex® C_18_ column (2.6 μm, 100 Å, 100 × 2.1 mm, Phenomenex, Aschaffenburg, Germany), applying a multistep gradient elution over a total run time of 8.25 min. The gradient for Mobile phase B (see Sect. 2.2.1 for composition of Mobile phases A and B) was programmed as follows: Starting at 25% and a flow rate of 0.45 mL/min, shifting to 70% at 6.5 min, and then to 90% at 6.8 min with flow rates increasing up to 0.60 mL/min. The gradient was rapidly returned to the initial conditions at 7.85 min, followed by re-equilibration. The injection volume was 10 µL for identifying all target analytes included in the method.

For the identification of further 5F-ADB metabolites, a second run was performed with 15 µL injection volume and the same LC-gradient, using previously published phase I and II metabolites for comparison [64, 65]. For a retention time comparison of the phase I metabolites, a 1 mg/mL stock solution of 5F-ADB was incubated into a pooled human liver microsome assay (pHLMs) according to a previously published protocol [66]. For comparison with true human metabolites, two serum samples from human cases that tested positive for 5F-ADB phase I and II metabolites, as well as an enzymatically hydrolyzed urine sample from a real human case with phase I metabolites were analysed, using previously published protocols [66–68]. In addition to retention time matching with products from the pHLMs and positive human samples, an unscheduled measurement was performed to detect other possible metabolites with the same transitions that may have formed in larvae. A summary of these metabolites, including several monohydroxylated derivatives and glucuronic acid conjugates, together with their mass transitions and optimised mass spectrometric parameters is provided in Table S2 of the Supporting information.

For quantification in larval samples, calibration curves for each analyte were generated using at least six calibration points in the calibration range 0.25–10 µg/kg (R^2^ ≥ 0.99). Chromatograms of the analytes at the lowest calibrator concentration are shown in Fig. S1 of the Supporting information. Calibration curves were generated from extracts of drug-free larvae that had been spiked with the deuterated internal standard substances and the synthetic cannabinoid analytes at the corresponding concentrations. Area ratios of the target ions between the analyte and the corresponding deuterated IS (Table S1) were plotted against substance concentration and Mandel’s *F* test (significance 99%) was applied to identify the linear range for each substance. Matrix effects and extraction efficiencies for each analyte were determined according to the post-extraction spike method [69], applying area ratios of target ions between the analyte and the corresponding deuterated IS at analyte concentrations in the middle of the calibration range (5 µg/kg). Matrix effects were calculated from area ratios of signals between analyte and the corresponding deuterated IS substance obtained from reconstitution solvent (Mobile phases A:B, 4:1) and extracted blank larval matrix that had been fortified to an analyte concentration of 5 µg/kg. Extraction efficiencies were estimated by comparing the signals in blank larval matrix fortified to 5 µg/kg with all compounds after extraction to the signal in samples fortified to the same concentration before extraction. The limits of detection (LOD) and limits of quantification (LOQ) for all SCRA analytes were determined from a dilution series in the concentration range between 0.025 µg/kg and 0.25 µg/kg in larval homogenate, followed by a calculation in validation software (Valistat ver. 2.0, Arvecon GmbH, Walldorf, Germany), considering a signal-to-noise ratio of at least 3.

## Results

### Effect of 5F-ADB on larval mass and length during development

**Fig. 5:**
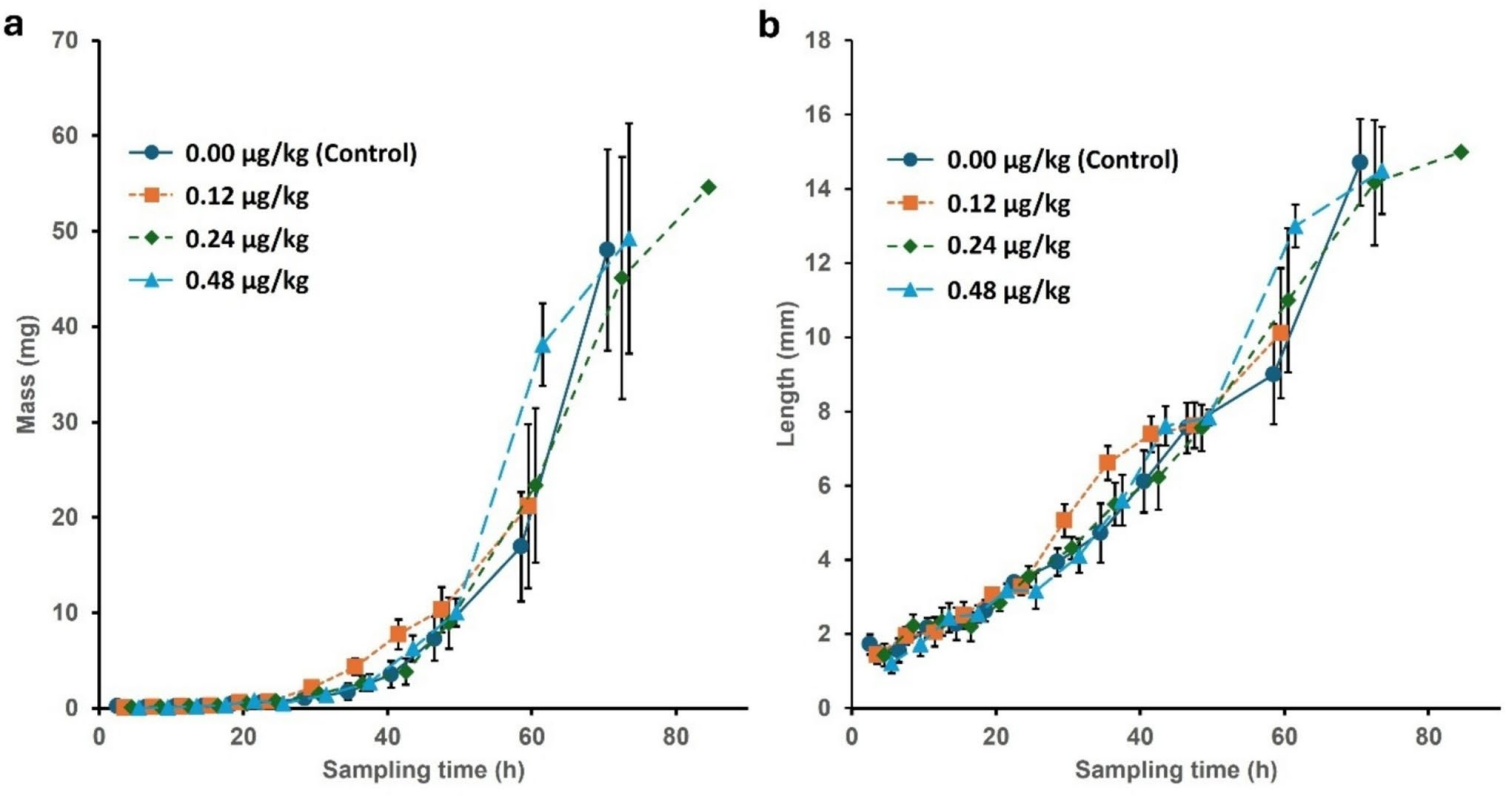
(**a**) Mass and (**b**) length as a function of sampling time (age) for larvae from the different 5F-ADB concentration treatments in In vitro experiment I. Each datapoint represents nine larvae, sampled equally from three replicate cups. Vertical bars denote 95% confidence intervals for the means (CI95)

The data from In vitro experiment I were truncated at 60 h because mortality in some treatments precluded further balanced sampling (Fig. 5). The end of exponential growth and the onset of the post-feeding phase occurred at about 72 h, so most of the growth period was represented in the data (Fig. 5). As may be expected of a growth process, the variances of the treatment cells were clearly heteroscedastic (Fig. 5), so the dependent variables were log-transformed before further analysis to homogenise the variances.

#### Effect of 5F-ADB on larval mass

The results of the ANOVA analysis for the effect of 5F-ADB dose, replicate beaker, and time are summarised in Supplementary Table S4. The beaker effect and its interactions were all not statistically significant, so the ANOVA was repeated without this variable to increase the power of the analysis of the other variables.

In the ANOVA (Table 2), the effect of sampling time (larval age) was highly significant, reflecting substantial changes in larval mass over time. Similarly, dose had a significant impact (Table 2), due to significant disparity between the control and the 1.2 µg/kg- and 4.8 µg/kg-treatments (Supplementary Fig. S2a). The interaction between time and dose was highly significant (Table 2), primarily due to sporadic high means in particular doses at different times, no clear overall dose-dependent pattern was obvious and size advantages at one sampling time vanished in subsequent samples (Fig. 5a). The most significant effect resulted from time (growth), followed by the interaction, and then the effect of dose. For example, larvae treated with 0.48 µg/kg of 5F-ADB were significantly heavier than those from all other treatment groups after 60 h of incubation, but were unremarkable at 72 h (Fig. 5a). Additionally, larvae treated with 0.24 µg/kg of 5F-ADB were significantly heavier than the control group at this time. The 0.12 µg/kg dose showed a trend towards higher values between 30 and 48 h of development, but these differences were not statistically significant (Fig. 5a).

**Table 2:** Two-way ANOVA, showing the effects of sampling time (larval age), 5F-ADB dose, and their interaction on *L. sericata* larval (log-transformed) mass

	SS	DF	MS	F	*p*	Partial eta-squared	Non-centrality	Observed power
Intercept	2.5875	1	2.58754	39.0375	0.000000	0.102451	39.038	0.999990
Time	231.5213	10	23.15213	349.2899	0.000000	0.910819	3492.899	1.000000
Dose	1.3090	3	0.43635	6.5831	0.000245	0.054594	19.749	0.971905
Time*Dose	4.8119	30	0.16040	2.4198	0.000078	0.175099	72.595	0.999844
Error	22.6689	342	0,06628					

SS: Sum of Squares, DF: Degrees of Freedom, MS: Mean Square, F: F-statistic, Significant effects (α = 0.05) are shown in bold.

#### Effect of 5F-ADB on larval length

- SS: Sum of Squares, DF: Degrees of Freedom, MS: Mean Square, F: F-statistic, Significant effects (α = 0.05) are indicated in bold type.

In the ANOVA (Table 3), time and dose both had significant effects on larval length, and their interaction was significant. Consistent with the analysis of larval mass, the largest effect on larval length was from time, *i.e.* larval growth, followed by the interaction and then dose, but the effect sizes of dose and the interaction were less marked (Tables 2 and 3). The control dose had significantly shorter larvae than the 1.2 µg/kg dose (Supplementary Fig. S2b), and as with mass, there was an erratic pattern of differences, but it was not entirely congruent with those shown by mass (Fig. 5b). For instance, the high mean mass seen at 0.48 µg/kg at 60 h was not coupled with an equally distinctive mean length (Fig. 5). Again, no overall dose-dependent tendency towards a pattern was evident except at 60 h; otherwise the dose curves were somewhat braided.

**Table 3:** Two-way ANOVA, showing the effects of sampling time (larval age) and 5F-ADB dose on *L. sericata* larval (log-transformed) length

	SS	DF	MS	F	*p*	Partial eta-squared	Non-centrality	Observed power
Intercept	120.0316	1	120.0316	11,899.03	0.000000	0.972061	11,899.03	1.000000
Time	26.5623	10	2.6562	263.32	0.000000	0.885049	2633.19	1.000000
Dose	0.1262	3	0.0421	4.17	0.006407	0.035291	12.51	0.852125
Time*Dose	0.6276	30	0.0209	2.07	0.001072	0.153923	62.22	0.998929
Error	3.4499	342	0.0101					

#### Toxicological analysis

Limits of detection (LODs), limits of quantification (LOQs), and linear ranges for all SCRA analytes and metabolites included in the method are summarised in Table 4. Matrix effects and extraction efficiencies are summarised in the supplementary Table S3.

**Table 4:** Limits of detection (LOD), limits of quantification (LOQ), and linear ranges calculated for 5F-ADB, ADB-BUTINACA, MDMB-4en-PINACA, and their metabolites in larvae

Analyte		LOD [µg/kg]	LOQ [µg/kg]	Linear Range [µg/kg]
5F-ADB		0.08	0.25	0.50–6.25
	5F-ADB hydrolysis product	0.06	0.14	0.50–6.25
	Hydrolytic/oxidative defluorination metabolite	0.03	0.05	0.25–8.75
ADB-BUTINACA		0.12	0.22	1.0–10.0
	ADB-BUTINACA 3,3-dimethylbutanoic acid metabolite	0.04	0.09	0.50–7.50
	ADB-BUTINACA *N*-(4-hydroxybutyl) metabolite	0.07	0.09	0.25–10.0
MDMB-4en-PINACA		0.05	0.14	0.25–7.50
	MDMB-4en-PINACA hydrolysis product	0.06	0.17	0.25–7.50

None of the larvae from In vitro experiment I survived until after 72 h of development. Of the larvae sampled during the actively feeding phase from In vitro experiment I, 5F-ADB was detectable in concentrations below the limit of quantification (*i.e.* < 0.25 µg/kg) in all samples, except for larvae from the 0 µg/kg-treatment, which tested negative. Not all (but most) actively feeding larval samples from In vitro experiment II, treated with 0.48 µg/kg and 1.8 µg/kg 5F-ADB tested positive for 5F-ADB. For all positive samples from the 0.48 µg/kg-treatment and some from the 1.8 µg/kg-treatment 5F-ADB was detected below the LOQ. All actively feeding larvae from the 7.7 µg/kg-treatment contained 5F-ADB concentrations above the LOQ, with an average concentration of 0.34 µg/kg. No clear effect of larval age on the detected drug concentration in the larvae was evident during the actively feeding stage. 5F-ADB was detectable in none of the post-feeding larvae from any of the treatments, except for one single sample from the 7.7 µg/kg-treatment, which contained only trace amounts of the substance.

None of the 5F-ADB metabolites, including the hydrolysis product, hydrolytic/oxidative defluorination derivative, and human or in vitro phase I and II metabolites was detected in any of the larval samples, irrespective of sampling time and treatment concentration.

## Discussion

### Effect of 5F-ADB on larval mass and length during development

To date, the literature on SCRAs has focused mainly on their pharmacology and toxicity in humans, as well as their detection in conventional biological matrices (*i.e.* human blood, urine, and rarely solid tissues) [15, 20, 32, 70, 71]. Several studies have demonstrated the association of molecules such as 5F-ADB with intoxication or death, underlining their role in forensic medicine [34, 36, 57, 59]. However, to date, no study has examined the impact of these substances on the development of necrophagous insects, notably *L. sericata*. Therefore, this study is the first to investigate the impact of a SCRA on the development of *L. sericata* and its potential implication for estimating the PMI_min_. This is a particularly important consideration, because 5F-ADB is still occasionally detected in drug-related deaths.

Our statistical outcomes indicate that time (*i.e.* growth) has the most significant impact on larval development, since larvae increase in mass and length as they continue to feed. However, the drug itself had no consistent effect on the growth of *L. sericata* larvae. Neither larval mass nor length was influenced by 5F-ADB concentrations that are typically found in peripheral blood of post-mortem cases. This finding is somewhat surprising, because other central nervous system depressing drugs are typically associated with inhibited insect development [72–76]. However, some authors also saw an opposite effect [77], indicating that the mechanisms involved in insect pharmacodynamics are complicated and not yet well-understood. Studies have shown that no cannabinoid receptors have been reported in flies in general [78], and specifically not in *L. sericata* [52] and the closely related blowfly species *Lucilia cuprina* (Weidemann, 1830) (Diptera: Calliphoridae) [79] in particular, which may (partially) explain our findings.

During entomological experiments, the potential impact of replicate number on experimental effect may skew the results of such studies [80]. Ideally, the experimental set-up should include adequate replication, taking into account insect mortality and possible human error. However, due to practical, financial, and time limitations, this goal is not always feasible. A high number of samples would require an even larger number of first-instar larvae at the beginning of the experiment, which would typically be associated with significant laborious effort [80]. Blowfly larvae grow better in feeding aggregations (apparently despite potential direct competition), so one may expect growth to be affected negatively as an aggregation is depleted by sampling and mortality. Another potential source of error is the measurement technique. Among the growth parameters, length is generally the most commonly applied measure for entomological investigations [81]. Larval length is measured using a microscope and a simple ruler, introducing the influence of *relative* error [14, 63, 81, 82], especially when larvae are very small. Perhaps because of the effect of relative error, in the graphs (Fig. 5) up to 24 h there are no significant differences in growth between different doses, which reduces the uncertainty associated with estimating PMIs from these measurements at those ages [14]. Mass measurements of very young larvae offer a more precise analysis technique. However, analytical balances with high precision are expensive and not always available to the analyst.

### Toxicological analysis of larvae exposed to 5F-ADB

In a comparative analysis on drugs detected in post-mortem human samples and larvae, Groth et al*.* [41] were able to identify some drugs (including the SCRAs AM-2201 and THJ-2201) in larvae, but not in any of the human specimens from the bodies they were sampled from. On the other hand, 5F-ADB was undetectable in larvae despite its presence in human hair and stomach contents. It was speculated that the negative findings could be partially attributed to the authors applying the same extraction method for larvae as for human specimens to purify the samples. As the larval matrix differs significantly in composition from most human specimens, it is likely that the application of the same extraction method to larvae would lead to the occurrence of significant matrix interferences and/or low extraction efficiencies. Resultantly, we explored the development and optimisation of pre-analytical methods for the detection of SCRAs, including 5F-ADB, from larvae.

Karampela et al*.* [83] successfully developed and validated a method for detecting the phytocannabinoid drug Δ^9^-tetrahydrocannabinol (Δ^9^-THC) and its human carboxylic acid metabolite (THC-COOH) in *L. sericata* larvae by LC–MS. However, thus far, no work on the detection of synthetic cannabinoid analogues from insect larvae has been reported. With this study, we are the first to present a method for detecting and quantifying SCRAs, including 5F-ADB, ADB-BUTINACA, MDMB-4en-PINACA, and their metabolites from necrophagous larvae with low limits of detection and quantification. Results obtained for matrix effects and extraction efficiencies for these substances underline the complexity of larvae as a toxicological matrix. Due to the presence of fat bodies, phospholipids in larvae may co-elute with lipophilic substances such as SCRAs, potentially leading to matrix interferences. Furthermore, despite our target analytes sharing a similar core structure, differences in functional groups (*e.g.* between parent compounds and their hydrolysis products) may cause substantial differences in chemical properties (*e.g.* polarity) and ionisation efficiency, which may impact both recovery from the matrix and signal response during mass spectrometric detection. In our study, matrix effects and recoveries of all hydrolysis products show unfavourable analytical quality. Nevertheless, their limits of detection and limits of quantification are in an acceptable range. Minimal matrix effects and promising extraction efficiencies were obtained for ADB-BUTINACA, its *N*-(4-hydroxybutyl) metabolite, and the hydrolytic/oxidative defluorination metabolite of 5F-ADB. The high ion suppression observed for 5F-ADB and MDMB-4en-PINACA could be compensated for by the good extraction efficiencies obtained with our method. Applying a different sample preparation technique may lead to less pronounced matrix effects for these two analytes, but recoveries (possibly also for some of the other analytes) would likely be reduced. The use of the same deuterated analogues as the analytes investigated would certainly allow optimal quantification of the analytes. However, due to the limited commercial availability of these substances, the most suitable deuterated SCRA analogues had to be applied. Nevertheless, in real forensic casework, the accurate quantification in larvae does not carry the same weight as for primary human specimens. Quantitative data from larvae cannot inform on the drug dose taken by the deceased, because no correlation between concentrations in larvae and the food source is to be expected [39, 41, 84]. It can thus be concluded that pre-analytical methods that would lead to good qualitative results would be beneficial in casework.

The phenomenon of no quantitative correlation was also observed in our in vitro experiments, during which larvae were fed with different concentrations of 5F-ADB and then analysed toxicologically. Our meta-analysis of over 120 fatalities reported in literature [24, 33, 36, 57–59] showed that 5F-ADB occurs in post-mortem cases at a mean concentration of 0.41 µg/L and a median concentration of 0.21 µg/L in peripheral blood (Fig. 3). Some post-mortem solid tissues contained less than 0.5 µg/kg of 5F-ADB (Table 1) [32, 60]. Resultantly, a concentration range of 0.12 µg/kg to 0.48 µg/kg 5F-ADB was chosen for our first in vitro study (Fig. 3). Based on the limited data available for post-mortem solid tissue concentrations (Table 1), a concentration range of 0.48–7.7 µg/kg was chosen for the second in vitro study. Most of the actively-feeding larvae that were exposed to typical post-mortem peripheral blood and low (0.12–0.48 µg/kg) and intermediate (1.8 µg/kg) solid tissue concentrations in the food medium contained small amounts of 5F-ADB, mostly below the limit of quantification (*i.e.* between 0.08 µg/kg and 0.25 µg/kg). Only larvae exposed to very high human solid tissue concentrations (*i.e.* 7.7 µg/kg) contained quantifiable levels of the drug in the lower concentration range of our calibration curve. These results are consistent with previous publications that state that only a small portion of a drug in the food medium is typically detectable in larvae [14, 39, 77, 84–87]. It also confirms that the analysis of larvae from a corpse cannot provide any reliable information about drug concentrations in the food source and thus also the dose taken by the deceased while alive [85, 86]. Furthermore, not all actively feeding larvae that were exposed to 5F-ADB contained detectable concentrations, which is in line with earlier findings that the absence of a drug in (some) larvae does not confirm its absence in the food source [41, 84]. Furthermore and in contrast to previous findings [14, 39], 5F-ADB concentrations in larvae could not be correlated with the developmental stage of actively feeding larvae. Nevertheless, actively feeding larvae could be useful for qualitative identification of 5F-ADB in a deceased person, especially in cases where human specimens are absent or highly putrefied. On the other hand, post-feeding larvae (*i.e.* larvae that have left the corpse in preparation for pupation) are unlikely to show a positive outcome. Larvae egest and excrete drugs before entering the post-feeding phase, resulting in a substantial decrease in drug levels in older larvae [14, 39, 88]. In cases where low concentrations are present in actively feeding larvae due to low concentrations in the food source (as is to be expected for 5F-ADB in real casework), no or only very small (likely undetectable) amounts would remain in larvae after excretion. Finally, we were unable to identify any 5F-ADB metabolites in any of the larval samples. Due to the relatively low concentrations of the parent drug in the food source and consequently in the larvae, it is unclear whether metabolites of 5F-ADB were undetectable in larvae because *L. sericata* is incapable of metabolising the drug, or whether metabolites were in fact present, but only at levels below the LODs.

Due to the high potency of 5F-ADB, even low doses of the drug can be associated with serious intoxications in humans and even deaths, resulting in relatively low detectable concentrations of the drug in post-mortem materials. However, low 5F-ADB concentrations in the corpse are generally not solely a result of the drug’s high potency. Several studies have highlighted the pronounced degradation of 5F-ADB under various conditions, leading to lower or even undetectable concentrations of the parent compound at the time of autopsy. In this regard, Krotulski et al. [89] demonstrated the drug’s high susceptibility to hydrolytic biotransformation in post-mortem blood, with approximately 90% of the compound degrading within seven days at room temperature, resulting in the corresponding acid (Fig. 1b). Due to the chemical lability of compounds like 5F-ADB that contain ester linkages, longer post-mortem intervals and the associated changes in pH, temperature, and microbial activity would lead to a higher degree of degradation [90, 91]. Since necrophagous larvae are typically associated with corpses already demonstrating signs of decomposition, it would be fair to assume that larvae would be exposed to 5F-ADB concentrations that are lower than was present in the body at the time of death. However, the demethylated analogue as hydrolysis product was detected neither in larvae from the first, nor the second in vitro study, which contained significantly higher drug concentrations in the food medium.

In a retrospective study of 43 post-mortem cases associated with 5F-ADB consumption, Boland et al*.* [59] reported a median concentration of 0.07 µg/L (range: 0.01–0.77 µg/L) for the parent compound in post-mortem peripheral blood, whereas the hydrolysis product showed a much higher median concentration of 15 µg/L (range: 2.0–110 µg/L). Similar concentration ratios between parent compound and hydrolysis product in post-mortem cases were observed by others [36, 58]. It is unknown whether the hydrolysis product may influence larval growth, whereas the primary aim of the current study was to investigate the influence of the parent compound alone. An in vivo study in mice has shown that the two metabolites included in the present study may contribute to the pharmacological activity of 5F-ADB, albeit not to the same extent as the parent compound [92]. Considering that 5F-ADB had no significant effect on larval growth, it is unlikely that the pharmacologically less active metabolites would. Pharmacological activity and interferences with larval growth is particularly not to be expected in the absence of cannabinoid receptors [52, 78, 79], although other mechanisms may also play a role [3].

### Study limitations

This study was limited to controlled conditions that do not fully replicate the fluctuating environmental variables, such as temperature, humidity, and microbial activity that would typically influence larval development in actual decomposition scenarios [74]. Furthermore, necrophagous blowfly species other than *L. sericata* may react differently to the drug, whereas this is not necessarily to be expected in the absence of cannabinoid receptors [52, 78, 79]. Furthermore, an in vitro set-up cannot consider ante-mortem pharmacokinetic processes in the human body. Nevertheless, the execution of experiments under controlled laboratory conditions with individual insect species are necessary before the effects can be evaluated under natural conditions. Applying an artificial food source facilitates the homogenous distribution of the toxicant under investigation throughout the matrix [8]. The true effect of a given drug concentration on insect development can thus be evaluated, which can provide fundamental data that could be applied in machine learning models to predict drug effects under natural conditions [93].

The calculated matrix effects and recoveries indicate that the toxicological method provides unfavourable analytical quality for some of the analytes, which may compromise quantitative detection of these substances in larvae. However, we could achieve low LODs and quantitative toxicological data from larvae are generally of little value for real casework because of the lack of correlations in concentrations with human specimens [8, 9, 85]. Conversely, the successful application of necrophagous larvae for the qualitative (or semi-quantitative) identification of drugs from a corpse may prove advantageous in forensic cases, particularly in instances where larvae are available, but standard toxicological specimens are not [38, 40].

## Conclusion

Our results show that 5F-ADB has no significant effect on the development of *L. sericata* larvae. This suggests that the presence of 5F-ADB in a corpse in our investigated concentration range does not have to be considered during PMI_min_ estimations when using *L. sericata* larvae.

In real forensic scenarios involving drugs that do, in fact, influence insect development, such considerations are generally subject to practical limitations [8, 62, 80, 94, 95]. Despite the knowledge of the effects that a particular drug may have on necrophagous larval growth, the incorporation of such knowledge during PMI_min_ estimations remains challenging for forensic entomologists. Firstly, close collaboration with forensic toxicologists is imperative to ascertain which substances were taken up by the insects. Furthermore, the combination of drugs in a corpse may result in different developmental alterations compared to the effects caused by the individual substance [93], as was demonstrated before for heroin and cocaine, and some antibiotics [75, 96]. Finally, given the inhomogeneous distribution of drugs in a corpse, concentration-dependent effects may be difficult to assess [8, 9]. These limitations emphasise the necessity for further research and improved cooperation between the two fields, before entomotoxicological aspects can be genuinely incorporated into PMI estimations of real forensic cases [9].

From a toxicological perspective, 5F-ADB could be identified in larvae that had been exposed to the substance, for example in the body of a deceased person who had ingested the substance before death. In their latest report on new psychoactive substances, the EUDA states that SCRAs continue to pose a public health threat, despite indications of a substantial decrease in their availability approximately two years ago [27]. The exploration of alternative methodologies for the post-mortem detection of SCRAs, such as 5F-ADB, particularly in cases of advanced stages of decomposition, thus has the potential to contribute to the elucidation of forensic cases. However, due to the high potency and chemical instability of 5F-ADB, post-mortem specimens typically contain only small levels of the drug. This may result in very low or undetectable concentrations in larvae, especially those that have already entered the post-feeding stage.

## Supplementary Information

Below is the link to the electronic supplementary material.

Supplementary file1 (DOCX 1310 KB)

## Data Availability

All data supporting the findings of this study are available within the paper and its Supplementary Information.
