# Supplementary material for: Entomological consequences and toxicological detection of synthetic cannabinoid receptor agonists (SCRAs) in necrophagous larvae (Diptera: Calliphoridae)
Source: Int J Legal Med. 2026 Jan 2;140(3):1349–65. doi: 10.1007/s00414-025-03688-8 (PMC13161333; doi:10.1007/s00414-025-03688-8)
Supplement: Supplementary file 1 — Supplementary file1 (DOCX 1310 KB) [file 414_2025_3688_MOESM1_ESM.docx]

**Supporting Information**

**Entomological consequences and toxicological detection of synthetic cannabinoid receptor agonists (SCRAs) in necrophagous larvae (Diptera: Calliphoridae)**

Chloé A. K. Blavier^a,b^, Martin H. Villet^c^, Annette Zschiesche^d,e^, Volker Auwärter^d^, Matthias Graw^a^, Christoph Geffert^f^, and Olwen C. Groth*^a^

^a^ Institute of Forensic Medicine, Ludwig-Maximilians-Universität in Munich, Nussbaumstrasse 26, D-80336 Munich, Germany

^b^ Polytech Nice Sophia Engineering School – University Côte d'Azur, 06903 Sophia Antipolis, France

^c^ Department of Zoology & Entomology, Rhodes University, Makhanda, 6140 South Africa

ORCID 0000-0002-4335-5667, *E-mail address*: [martin.villet@gmail.com](mailto:martin.villet@gmail.com)

^d^ Institute of Forensic Medicine, Forensic Toxicology, Medical Center – University of Freiburg, Faculty of Medicine, University of Freiburg, Albertstrasse 9, 79104 Freiburg, Germany, ORCID [0000-0002-1883-2804](https://orcid.org/0000-0002-1883-2804)

^e^ Hermann Staudinger Graduate School, University of Freiburg, Hebelstrasse 27, 79104 Freiburg, Germany, ORCID 0009-0005-9017-0111

^f^ Labor Staber, Bremer Strasse 9, 01665 Klipphausen, Germany

*Corresponding author

ORCID 0000-0003-4672-041X, *E-mail address*: [olwen.groth@med.uni-muenchen.de](mailto:olwen.groth@med.uni-muenchen.de)

**Table S1.** Transitions and optimised mass spectrometric parameters used in Multiple Reaction Monitoring (MRM) mode for SCRA analytes and deuterated analogues. Analytes are followed by their corresponding deuterated analogues (bold type)

| **Analyte** | | **RT**  **[min]** | **Q1**  **[Da]** | **Q3**  **[Da]** | **DP**  **[V]** | **EP**  **[V]** | **CE**  **[V]** | **CXP**  **[V]** |
| --- | --- | --- | --- | --- | --- | --- | --- | --- |
|  | ADB-BUTINACA *N*-(4-hydroxy-butyl) metabolite | 2.16 | 347.20 | 217.10 | 50 | 10 | 35 | 17 |
|  |  |  |  | 302.20 | 50 | 10 | 18 | 18 |
|  | **AB-PINACA *N*-pentanoic acid metabolite-*d_4_*** | **2.00** | **365.11** | **320.10** | **64** | **10** | **21** | **32** |
|  | 5F-ADB oxidative/hydrolytic defluorination metabolite | 4.35 | 376.20 | 213.00 | 62 | 10 | 37 | 15 |
|  |  |  |  | 316.00 | 62 | 10 | 24 | 19 |
|  | **JWH-250 *N*-(4-hydroxypentyl) metabolite-*d_5_*** | **4.24** | **357.20** | **121.10** | **72** | **10** | **31** | **20** |
|  | 5F-ADB hydrolysis product | 4.75 | 364.00 | 233.00 | 50 | 10 | 33 | 15 |
|  |  |  |  | 318.30 | 50 | 10 | 20 | 15 |
|  | ADB-BUTINACA 3,3-dimethylbutanoic acid metabolite | 5.14 | 332.20 | 201.10 | 50 | 10 | 35 | 17 |
|  |  |  |  | 145.00 | 50 | 10 | 57 | 14 |
|  | MDMB-4en-PINACA  butanoic acid metabolite | 5.21 | 344.00 | 213.00 | 70 | 10 | 35 | 15 |
|  |  |  |  | 298.00 | 70 | 10 | 20 | 10 |
|  | **JWH-073 *N*-(4-hydroxybutyl) metabolite-*d_5_*** | **4.42** | **349.20** | **155.12** | **100** | **10** | **37** | **12** |
| ADB-BUTINACA | | 4.47 | 331.20 | 201.10 | 55 | 10 | 35 | 15 |
|  |  |  |  | 286.19 | 55 | 10 | 21 | 15 |
| **AB-PINACA-*d_9_*** | | **4.45** | **340.27** | **224.17** | **85** | **10** | **34** | **15** |
| 5F-ADB | | 6.15 | 378.10 | 233.20 | 70 | 10 | 35 | 11 |
|  |  |  |  | 318.30 | 70 | 10 | 24 | 12 |
| MDMB-4en-PINACA | | 6.69 | 358.00 | 213.00 | 70 | 10 | 35 | 15 |
|  |  |  |  | 298.20 | 70 | 10 | 25 | 17 |
| **JWH-015-*d_7_*** | | **6.57** | **335.20** | **155.10** | **85** | **5** | **34** | **11** |

RT: Retention time, Q1: *m/z* of precursor ion, Q3: *m/z* of fragment ion, DP: Declustering Potential, EP: Entrance Potential, CE: Collision energy, CXP: Collision cell exit potential

**Table S2.** Transitions and optimised mass spectrometric parameters used in MRM mode to detect 5F-ADB phase I metabolites and glucuronic acid conjugates, including those measured in unscheduled mode (RT = *Unsch.*), together with those of the deuterated internal standard analogues

| **Analyte** | **RT [min]** | **Q1 [Da]** | **Q3**  **[Da]** | **DP**  **[V]** | **EP**  **[V]** | **CE**  **[V]** | **CXP [V]** |
| --- | --- | --- | --- | --- | --- | --- | --- |
| 5F-ADB 1 | 6.15 | 378.1 | 233.2 | 70 | 10 | 35 | 11 |
| 5F-ADB 2 | 6.15 | 378.1 | 318.3 | 70 | 10 | 24 | 12 |
| 5F-ADB 3 | 6.15 | 378.1 | 145 | 70 | 10 | 55 | 10 |
| 5F-ADB hydrolysis 1 | 4.75 | 364.0 | 233.001 | 50 | 10 | 32 | 15 |
| 5F-ADB hydrolysis 2 | 4.75 | 364 | 318 | 50 | 10 | 20 | 15 |
| 5F-ADB hydrolysis 3 | 4.75 | 364.00 | 145.001 | 50 | 10 | 52 | 15 |
| 5F-ADB *N*-5-OH 1 | 4.35 | 376.2 | 213 | 62 | 10 | 37 | 15 |
| 5F-ADB *N*-5-OH 2 | 4.35 | 376.2 | 231 | 62 | 10 | 33 | 17 |
| 5F-ADB *N*-5-OH 3 | 4.35 | 376.2 | 316 | 62 | 10 | 24 | 19 |
| 5F-ADB *N*-5-OH 4 | 4.35 | 376.2 | 145 | 62 | 10 | 52 | 14 |
| MDMB-INACA 1 | 3.75 | 290.15 | 230.1 | 48 | 10 | 26 | 15 |
| MDMB-INACA 2 | 3.75 | 290.15 | 145 | 48 | 10 | 47 | 11 |
| 5F-ADB *N*-pentanoic acid 1 | *Unsch.* | 390.2 | 217.1 | 68 | 10 | 39 | 13 |
| 5F-ADB *N*-pentanoic acid 2 | *Unsch.* | 390.2 | 227.1 | 68 | 10 | 37 | 14 |
| 5F-ADB *N*-pentanoic acid 3 | *Unsch.* | 390.2 | 245.1 | 68 | 10 | 35 | 15 |
| 5F-ADB *N*-pentanoic acid 4 | *Unsch.* | 390.2 | 330.2 | 68 | 10 | 26 | 19 |
| 5F-ADB *N*-pentanoic acid 5 | *Unsch.* | 390.2 | 145 | 68 | 10 | 56 | 11 |
| 5F-ADB-OH 1 | *Unsch.* | 394.2 | 249.1 | 62 | 10 | 34 | 15 |
| 5F-ADB-OH 2 | *Unsch.* | 394.2 | 231.1 | 62 | 10 | 36 | 14 |
| 5F-ADB-OH 3 | *Unsch.* | 394.2 | 334.2 | 62 | 10 | 26 | 18 |
| 5F-ADB-OH 4 | *Unsch.* | 394.2 | 145 | 62 | 10 | 55 | 12 |
| 5F-ADB-OH 5 | *Unsch.* | 394.2 | 161 | 62 | 10 | 53 | 13 |
| 5F-ADB-OH 6 | *Unsch.* | 394.2 | 213.1 | 62 | 10 | 38 | 14 |
| 5F-ADB-OH 7 | *Unsch.* | 394.2 | 233.2 | 62 | 10 | 35 | 15 |
| 5F-ADB hyd + OH 1 | *Unsch.* | 380.2 | 249.1 | 61 | 10 | 35 | 15 |
| 5F-ADB hyd + OH 2 | *Unsch.* | 380.2 | 231.1 | 61 | 10 | 37 | 14 |
| 5F-ADB hyd + OH 3 | *Unsch.* | 380.2 | 334.2 | 61 | 10 | 24 | 17 |
| 5F-ADB hyd + OH 4 | *Unsch.* | 380.2 | 145 | 61 | 10 | 53 | 12 |
| 5F-ADB hyd + OH 5 | *Unsch.* | 380.2 | 161 | 61 | 10 | 52 | 13 |
| 5F-ADB hyd + OH 6 | *Unsch.* | 380.2 | 213.1 | 61 | 10 | 39 | 14 |
| 5F-ADB hyd + OH 7 | *Unsch.* | 380.2 | 233.2 | 61 | 10 | 37 | 15 |
| 5F-ADB hyd + *N*-5-OH 1 | *Unsch.* | 362.21 | 231.1 | 60 | 10 | 34 | 14 |
| 5F-ADB hyd+ *N*-5-OH 2 | *Unsch.* | 362.21 | 316.2 | 60 | 10 | 23 | 15 |
| 5F-ADB hyd+ *N*-5-OH 3 | *Unsch.* | 362.21 | 145 | 60 | 10 | 52 | 12 |
| 5F-ADB hyd + acid 1 | *Unsch.* | 376.18 | 217.1 | 65 | 10 | 38 | 13 |
| 5F-ADB hyd + acid 2 | *Unsch.* | 376.18 | 227.1 | 65 | 10 | 36 | 14 |
| 5F-ADB hyd + acid 3 | *Unsch.* | 376.18 | 245.1 | 65 | 10 | 34 | 15 |
| 5F-ADB hyd + acid 4 | *Unsch.* | 376.18 | 145 | 65 | 10 | 56 | 11 |
| 5F-ADB hyd + acid 5 | *Unsch.* | 376.18 | 330.2 | 65 | 10 | 21 | 18 |
| 5F-ADB hyd-H2 1 | *Unsch.* | 362.2 | 213.1 | 62 | 10 | 37 | 12 |
| 5F-ADB hyd-H2 2 | *Unsch.* | 362.2 | 233.1 | 62 | 10 | 34 | 14 |
| 5F-ADB hyd-H2 3 | *Unsch.* | 362.2 | 318.2 | 62 | 10 | 21 | 16 |
| 5F-ADB hyd-H2 4 | *Unsch.* | 362.2 | 145 | 62 | 10 | 55 | 11 |
| 5F-ADB hyd +Gluc 1 | *Unsch.* | 540.2 | 540.2 | 80 | 10 | 5 | 18 |
| 5F-ADB hyd +Gluc 2 | *Unsch.* | 540.2 | 364.2 | 80 | 10 | 29 | 16 |
| 5F-ADB hyd +Gluc 3 | *Unsch.* | 540.2 | 233.1 | 80 | 10 | 42 | 12 |
| 5F-ADB hyd +Gluc 4 | *Unsch.* | 540.2 | 318.2 | 80 | 10 | 31 | 14 |
| 5F-ADB hyd +Gluc 5 | *Unsch.* | 540.2 | 213.1 | 80 | 10 | 45 | 11 |
| 5F-ADB hyd +Gluc 6 | *Unsch.* | 540.2 | 145 | 80 | 10 | 68 | 10 |
| 5F-ADB OH + Gluc 1 | *Unsch.* | 570.25 | 570.246 | 80 | 10 | 5 | 18 |
| 5F-ADB OH + Gluc 2 | *Unsch.* | 570.25 | 145 | 80 | 10 | 69 | 11 |
| 5F-ADB OH + Gluc 3 | *Unsch.* | 570.25 | 231.1 | 80 | 10 | 47 | 12 |
| 5F-ADB OH + Gluc 4 | *Unsch.* | 570.25 | 249.1 | 80 | 10 | 45 | 13 |
| 5F-ADB OH + Gluc 5 | *Unsch.* | 570.25 | 334.2 | 80 | 10 | 38 | 14 |
| 5F-ADB OH + Gluc 6 | *Unsch.* | 570.25 | 394.2 | 80 | 10 | 28 | 16 |
| 5F-ADB OH + Gluc 7 | *Unsch.* | 570.25 | 161 | 80 | 10 | 67 | 11 |
| 5F-ADB *N*-5-OH + Gluc 1 | *Unsch.* | 552.26 | 552.255 | 80 | 10 | 5 | 17 |
| 5F-ADB *N*-5-OH + Gluc 2 | *Unsch.* | 552.26 | 145 | 80 | 10 | 67 | 11 |
| 5F-ADB *N*-5-OH + Gluc 3 | *Unsch.* | 552.26 | 213 | 80 | 10 | 47 | 12 |
| 5F-ADB *N*-5-OH + Gluc 4 | *Unsch.* | 552.26 | 231 | 80 | 10 | 45 | 13 |
| 5F-ADB *N*-5-OH + Gluc 5 | *Unsch.* | 552.26 | 376.2 | 80 | 10 | 28 | 15 |
| 5F-ADB *N*-5-OH + Gluc 6 | *Unsch.* | 552.26 | 316.2 | 80 | 10 | 36 | 14 |
| 5F-ADB acid + Gluc 1 | *Unsch.* | 566.23 | 566.234 | 80 | 10 | 5 | 17 |
| 5F-ADB acid + Gluc 2 | *Unsch.* | 566.23 | 390.2 | 80 | 10 | 27 | 15 |
| 5F-ADB acid + Gluc 3 | *Unsch.* | 566.23 | 217 | 80 | 10 | 47 | 12 |
| 5F-ADB acid + Gluc 4 | *Unsch.* | 566.23 | 227 | 80 | 10 | 45 | 13 |
| 5F-ADB acid + Gluc 5 | *Unsch.* | 566.23 | 245 | 80 | 10 | 43 | 14 |
| 5F-ADB acid + Gluc 6 | *Unsch.* | 566.23 | 145 | 80 | 10 | 68 | 11 |
| 5F-ADB acid + Gluc 7 | *Unsch.* | 566.23 | 330.2 | 80 | 10 | 34 | 16 |
| 5F-ADB acid + hyd + Gluc 1 | *Unsch.* | 552.22 | 552.219 | 80 | 10 | 5 | 17 |
| 5F-ADB acid + hyd + Gluc 2 | *Unsch.* | 552.22 | 217 | 80 | 10 | 47 | 12 |
| 5F-ADB acid + hyd + Gluc 3 | *Unsch.* | 552.22 | 227 | 80 | 10 | 45 | 13 |
| 5F-ADB acid + hyd + Gluc 4 | *Unsch.* | 552.22 | 245 | 80 | 10 | 43 | 14 |
| 5F-ADB acid + hyd + Gluc 5 | *Unsch.* | 552.22 | 376.2 | 80 | 10 | 28 | 16 |
| 5F-ADB acid + hyd + Gluc 6 | *Unsch.* | 552.22 | 145 | 80 | 10 | 69 | 11 |
| 5F-ADB acid + hyd + Gluc 7 | *Unsch.* | 552.22 | 330.2 | 80 | 10 | 35 | 15 |
| 5F-ADB *N*-5-OH + hyd + Gluc 1 | *Unsch.* | 538.24 | 538.24 | 80 | 10 | 5 | 18 |
| 5F-ADB *N*-5-OH + hyd + Gluc 2 | *Unsch.* | 538.24 | 145 | 80 | 10 | 67 | 11 |
| 5F-ADB *N*-5-OH + hyd + Gluc 3 | *Unsch.* | 538.24 | 213 | 80 | 10 | 48 | 13 |
| 5F-ADB *N*-5-OH + hyd + Gluc 4 | *Unsch.* | 538.24 | 231 | 80 | 10 | 46 | 14 |
| 5F-ADB *N*-5-OH + hyd + Gluc 5 | *Unsch.* | 538.24 | 362.21 | 80 | 10 | 28 | 16 |
| 5F-ADB *N*-5-OH + hyd + Gluc 6 | *Unsch.* | 538.24 | 316.2 | 80 | 10 | 35 | 15 |
| 5F-ADB Hyd + OH + Gluc 1 | *Unsch.* | 556.23 | 556.231 | 80 | 10 | 5 | 18 |
| 5F-ADB Hyd + OH + Gluc 2 | *Unsch.* | 556.23 | 145 | 80 | 10 | 68 | 11 |
| 5F-ADB Hyd + OH + Gluc 3 | *Unsch.* | 556.23 | 231 | 80 | 10 | 47 | 13 |
| 5F-ADB Hyd + OH + Gluc 4 | *Unsch.* | 556.23 | 249 | 80 | 10 | 45 | 14 |
| 5F-ADB Hyd + OH + Gluc 5 | *Unsch.* | 556.23 | 161 | 80 | 10 | 67 | 12 |
| 5F-ADB Hyd + OH + Gluc 6 | *Unsch.* | 556.23 | 334.2 | 80 | 10 | 36 | 15 |
| 5F-ADB Hyd + OH + Gluc 7 | *Unsch.* | 556.23 | 380.2 | 80 | 10 | 28 | 16 |

RT: Retention time, Q1: *m/z* of precursor ion, Q3: *m/z* of fragment ion, DP: Declustering Potential, EP: Entrance Potential, CE: Collision energy, CXP: Collision cell exit potential

Gluc: glucuronic acid conjugate, Hyd: hydrolysis product; OH: monohydroxylation product; *Unsch.*: measured in unscheduled mode


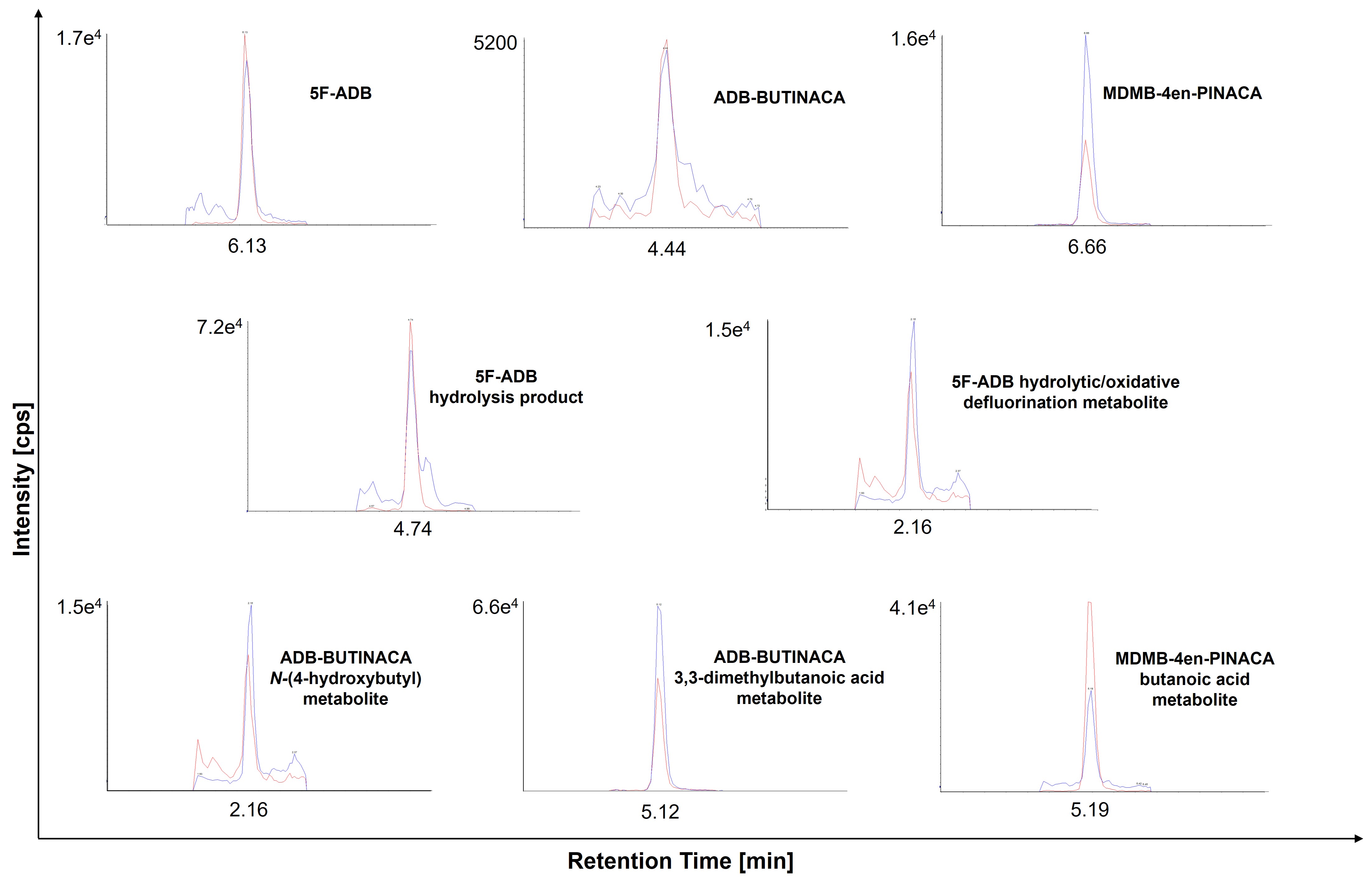
**Fig. S1.** Chromatograms for the target and qualifier ions for each of the analytes depicted in Fig. S1 at a concentration of 0.25 µg/kg

**Table S3.** Matrix effects and extraction efficiencies, calculated according to the post-extraction addition method [1] at 5 µg/kg

| **Analyte** | | **Matrix**  **Effects*** | **Extraction**  **Efficiencies** |
| --- | --- | --- | --- |
| 5F-ADB | | 10.9% | 71.3% |
|  | 5F-ADB hydrolysis product | *Significant ion enhancement* | 44.6% |
|  | Hydrolytic/oxidative defluorination metabolite | 118.3% | 51.0% |
| ADB-BUTINACA | | 104.1% | 97.0% |
|  | ADB-BUTINACA 3,3-dimethylbutanoic acid metabolite | *Significant ion enhancement* | 37.4% |
|  | ADB-BUTINACA *N*-(4-hydroxybutyl) metabolite | 92.2% | 90.4% |
| MDMB-4en-PINACA | | 22.4% | 83.4% |
|  | MDMB-4en-PINACA hydrolysis product | *Significant ion enhancement* | 42.5% |

*Values above 100% are indicative of ion enhancement and values below 100% of ion suppression

**Table S4** Three-Way ANOVA, showing the effects of time, 5F-ADB dose, and beaker on larval mass

|  | SS | DF | MS | F | *p* |
| --- | --- | --- | --- | --- | --- |
| Intercept | **6326.73** | **1** | **6326.727** | **613.7626** | **0.000000** |
| Time | **17439.99** | **10** | **1743.999** | **169.1872** | **0.000000** |
| Dose | **336.59** | **3** | **112.196** | **10.8842** | **0.000001** |
| beaker nr | 28.72 | 2 | 14.361 | 1.3931 | 0.250180 |
| time*dose | **2081.67** | **30** | **69.389** | **6.7315** | **0.000000** |
| time*beaker nr | 261.21 | 20 | 13.061 | 1.2670 | 0.201463 |
| dose*beaker nr | 119.76 | 6 | 19.960 | 1.9363 | 0.075434 |
| time*dose*beaker nr | 769.85 | 60 | 12.831 | 1.2447 | 0.126566 |
| Error | 2628.57 | 255 | 10.308 |  |  |

SS: Sum of Squares, DF: Degrees of Freedom, MS: Mean Square, F: F-statistic, Significant effects (*p* < 0.05) are indicated in bold type

**Table S5** Three-Way ANOVA, showing the effects of sampling time (larval age) and 5F-ADB dose on larval length

|  | SS | DF | MS | F | *p* |
| --- | --- | --- | --- | --- | --- |
| Intercept | **7592.636** | **1** | **7592.636** | **9571.489** | **0.000000** |
| beaker nr | 3.379 | 2 | 1.689 | 2.130 | 0.120973 |
| Time | **2791.236** | **10** | **279.124** | **351.871** | **0.000000** |
| Dose | **25.368** | **3** | **8.456** | **10.660** | **0.000001** |
| beaker nr*time | 19.685 | 20 | 0.984 | 1.241 | 0.220955 |
| beaker nr*dose | 9.423 | 6 | 1.571 | 1.980 | 0.068948 |
| time*dose | **96.657** | **30** | **3.222** | **4.062** | **0.000000** |
| beaker nr*time*dose | 63.934 | 60 | 1.066 | 1.343 | 0.061965 |
| Error | 203.867 | 257 | 0.793 |  |  |

SS: Sum of Squares, DF: Degrees of Freedom; MS: Mean Square, F: F-statistic, Significant effects (*p* < 0.05) are shown in bold type

**
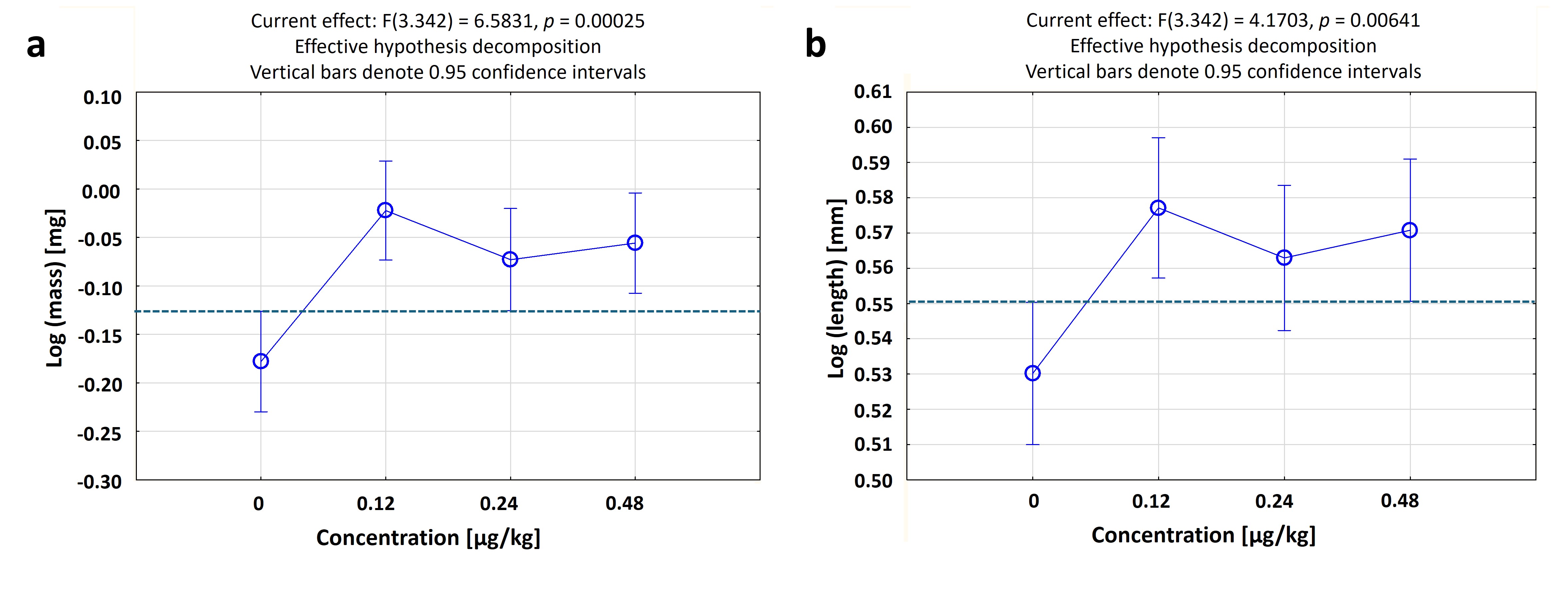
**

**Fig. S2. (a)** Log-transformed mean masses and **(b)** log-transformed mean lengths of larvae in the four dose treatments of *In vitro* Experiment I

**References**

1. Matuszewski BK, Constanzer ML, Chavez-Eng CM (2003) Strategies for the assessment of matrix effect in quantitative bioanalytical methods based on HPLC-MS/MS. Anal Chem 75:3019–3030
